# Hybrid Polyester-Hydrogel Electrospun Scaffolds for Tissue Engineering Applications

**DOI:** 10.3389/fbioe.2019.00231

**Published:** 2019-09-25

**Authors:** Ana Rita Gonçalves de Pinho, Ines Odila, Anne Leferink, Clemens van Blitterswijk, Sandra Camarero-Espinosa, Lorenzo Moroni

**Affiliations:** ^1^Tissue Regeneration Department, Institute for BioMedical Technology and Technical Medicine, University of Twente, Enschede, Netherlands; ^2^MERLN Institute for Technology-inspired Regenerative Medicine, Complex Tissue Regeneration Department, Maastricht University, Maastricht, Netherlands

**Keywords:** electrospinning, blended polymers, scaffolds, alginate, tissue engineering

## Abstract

Electrospinning is an attractive fabrication process providing a cost-effective and straightforward technic to make extra-cellular matrix (ECM) mimicking scaffolds that can be used to replace or repair injured tissues and organs. Synthetic polymers as poly (ε-caprolactone) (PCL) and poly (ethylene oxide terephthalate)-poly(butylene terephthalate) (PEOT/PBT) have been often used to produce scaffolds due to their good processability, mechanical properties, and suitable biocompatibility. While synthetic polymers can mimic the physical features of native ECM, natural polymers like alginate are better suited to recapitulate its hydrated state or introduce functional groups that are recognized by cells (e.g., –NH_2_). Thus, this study aims at creating electrospun meshes made of blended synthetic and natural polymers for tissue engineering applications. Polyethylene oxide (PEO), PCL, and PEOT/PBT were used as a carrier of Alginate. Scaffolds were electrospun at different flow rates and distances between spinneret and collector (air gap), and the resulting meshes were characterized in terms of fiber morphology, diameter, and mesh inter-fiber pore size. The fiber diameter increased with increasing flow rate, while there was no substantial influence of the air gap. On the other hand, the mesh pore size increased with increasing air gap, while the effect of flow rate was not significant. Cross-linking and washing of alginate electrospun scaffolds resulted in smaller fiber diameter. These newly developed scaffolds may find useful applications for tissue engineering strategies as they resemble physical and chemical properties of tissue ECM. Human Dermal Fibroblasts were cultured on PCL and PCL/Alginate scaffolds in order to create a dermal substitute.

## Introduction

The paradigm of tissue engineering is the regeneration of tissues and organs using cells and biomaterial-based approaches alone or in combination, for the replacement, or repair of damaged native tissues. To engineer living tissues in biomaterial-based strategies, biodegradable scaffolds are an important element providing the cells with an environment necessary for seeding, proliferation, invasion, and differentiation until defining the final shape of the regenerated tissue (Hutmacher, 2000; Zhang et al., 2007). Scaffolds must be (i) porous to allow cell growth and flow transport of nutrients and metabolic waste; (ii) bioresorbable and biocompatible with controllable degradation or resorption rates to match the rate of tissue regeneration; (iii) have adequate surface chemistry for cell attachment and formation of new tissue; (iv) and suitable mechanical properties to match those of the tissues at the site of implantation (Kim and Mooney, 1998; Hutmacher et al., 2001).

Electrospinning (ESP) is an electrostatically driven technology able to produce scaffolds that can mimic the architecture (geometry, morphology, and/or topography) as well as physico-chemical properties of extracellular matrix (ECM) in a simple manner with reduced associated costs (Mengyan Li et al., 2005). It is a highly flexible method of producing continuous fibers with diameters within the micron to sub-micron range out of a wide range of materials comprising natural and synthetic polymers, composites and ceramics (Cao et al., 2009). Due to the versatility of this technique, electrospun scaffolds have already been applied in several areas of tissue engineering including cardiovascular (Heydarkhan-Hagvall et al., 2008), musculoskeletal (Li et al., 2007), and neural tissue engineering (Yang et al., 2004; Nisbet et al., 2007), as well as to study how to control stem cell activity (Li et al., 2005; Suwantong et al., 2007).

The size and microstructure of the fibers comprising an electrospun scaffold are dependent on several parameters related to the intrinsic properties of the initial polymer solution to be spun and to the processing conditions. Namely, the fabrication process can be tailored by varying on one side the type of polymer and solvent, solution concentration, electrical conductivity, and surface tension, and on the other side the type of collector, the applied voltage to the solution, the distance between the spinneret and collector (air gap) and the flow rate (Ladd et al., 2014). It is also possible to incorporate many different types of molecules during the electrospinning fabrication process to generate functionalized nanofibers (Beachley and Wen, 2010). Each of these variables, in addition to temperature and humidity of the surrounding environment, can significantly influence the physical properties of the resulting fibrillar meshes (Li and Xia, 2004; Subbiah et al., 2005). When the biomaterial of choice is selected, the flow rate and air gap may also play an essential role in determining the characteristics of the obtained scaffolds (Moroni et al., 2006). Considering the flow rate, which influences the jet material and the material transfer rate, it is known that lower flow rates are more desirable as the solvent will have more time to evaporate (Yuan et al., 2004). According to Zong et al., the fiber and mesh pore diameter increase when the flow rate increases for different materials (Zong et al., 2002). This knowledge has been corroborated by other studies (Zhang et al., 2005; Zuo et al., 2005). However, high flow rates may generate beaded fibers due to the inability of solvent evaporation before reaching the collector (Wannatong et al., 2004; Yuan et al., 2004; Zuo et al., 2005). The same can be observed when the air gap is too large (Geng et al., 2005; Ki et al., 2005), thus implying that a minimum distance between the spinneret and the collector is required for homogenous fiber formation (Jalili and Hosseini, 2005; Ki et al., 2005). Also, studies with different polymers demonstrated that the effect of varying the air gap on fiber diameter is not as significant as other parameters previously described (Zhang et al., 2005; Zhao et al., 2005).

In the last few years, several biomaterials have been used to obtain electrospun micro- and nanofibers. Synthetic biodegradable polymers are still the most appealing biomaterials to construct scaffolds due to their well-known excellent processability and mechanical performance since they can be processed into desired shapes with mechanical strength and degradation rate matching the newly forming tissue (Chew et al., 2006; Murugan and Ramakrishna, 2007). However, these synthetic nanofibers do not possess cell recognition sites, leading to reduced cell affinity. The surfaces of the scaffolds are relatively hydrophobic, which may restrain cell seeding and lead to unspecific cellular activity. On the other hand, naturally derived polymers have shown good cell interaction and a more hydrophilic surface associated with enhanced cell seeding efficiency. Yet, the scaffolds constructed with these materials are mechanically weak and unstable to maintain desired shapes (Chen et al., 2000, 2004; Li et al., 2002; Zhang et al., 2007). In order to combine the advantages of both materials, naturally derived polymers are often chemically modified via crosslinking or blended with other natural or synthetic biomaterials to improve their physical and biological properties, thus possibly increasing their regenerative efficacy (Chen et al., 2000; Mengyan Li et al., 2005).

Electrospun nanofibers are known to have a great capability for wound healing and skin tissue regeneration (Khil et al., 2003; Jayarama Reddy et al., 2013; Kataria et al., 2014). Over the past decade, most research in this field has emphasized the use of biodegradable and biocompatible polymers as they can be easily washed off the wound surface (Kong et al., 2009). Alginate, a biodegradable polymer derived from seaweed, is structurally similar to glycosaminoglycans (GAGs) that are one of the major components of ECM in human tissue. It is usually used in the form of a hydrogel upon crosslinking with divalent cations in numerous applications like wound healing, drug delivery and tissue engineering of skin, cartilage, bone, or liver (Yang et al., 2001; Paul and Sharma, 2004; Li and Zhang, 2005; Rani et al., 2011; Lee and Mooney, 2012).

Skin is mainly composed of three layers: epidermis, dermis, and hypodermis. The complex nature of wound healing requires the migration and proliferation of keratinocytes and fibroblasts (Jayarama Reddy et al., 2013) that are the primary cells present in the dermis. They are known to affect keratinocyte proliferation (in the epidermis) and to be related to some immune responses of skin (Tuan et al., 1994; Bouffi et al., 2011). Additionally, researchers have previously recognized improved wound healing after the seeding of fibroblasts into a dermal substitute (Coulomb et al., 1998; Ojeh et al., 2001).

The treatment of acute and chronic wounds is a pressing need, and alginate-based wound dressings offer many advantages in comparison to the traditional wound dressings (e.g., gauze). The traditional methods work basically as a barrier, keeping the wound dry by allowing evaporation of wound exudates while preventing the entry of the pathogen into the wound (Boateng et al., 2008). The choice of alginate arose from its suitable characteristics: highly absorbent, preventing both accumulation of wound exudate and wound dehydration, as well as human fibroblasts stimulation (Shamshina et al., 2014). Furthermore, alginate dressings maintain a physiologically moist microenvironment that promotes rapid epithelialization, healing, and the formation of granulation tissue (Lee and Mooney, 2012). Various alginate dressings including AlgicellTM (Derma Sciences) AlgiSite MTM (Smith & Nephew), Comfeel PlusTM (Coloplast), KaltostatTM (ConvaTec), SorbsanTM (UDL Laboratories), and TegagenTM (3M Healthcare) are commercially available (Lee and Mooney, 2012). Moreover, numerous papers have suggested that certain alginate dressings (e.g., Kaltostat®) can enhance wound healing by making produce higher levels of cytokines such as interleukin-6 and tumor necrosis factor-α (Thomas et al., 2000), resulting in pro-inflammatory factors that are beneficial for wound healing (Murakami et al., 2010).

The electrospinning of alginate solution remains challenging (Bhattarai et al., 2006). A key issue is the control of the sol-gel transition because the gelation of alginate solutions starts at very low polymer concentrations (~2 wt.%) when in presence of divalent cations. Below this threshold, the solution does not contain enough solid mass to produce continuous fibers, thus resulting in a beaded structure. While at higher values, it is so viscous that electrostatic forces are not able to extrude it (Kataria et al., 2014). Research has been carried out on how to reduce the solution viscosity, by which gelation can occur at a higher polymer concentration (Çaykara et al., 2005).

A possible route to improve alginate electrospinning could be to hybridize it with a synthetic carrier. The aim of this study was, therefore, to create hybrid scaffolds comprised of synthetic and natural polymers, thus offering a more easy and cost-effective route for modifying and tailoring material properties. Different ways to overcome this problem and create fibers containing alginate were compared. While alginate blending with poly (ethylene oxide) (PEO) has shown to facilitate the spinning process, this is a water soluble polymer with low mechanical properties and only short-term stability (Hutmacher, 2000). Thus, alginate electrospun scaffolds were fabricated using PEO as a carrier, while polycaprolactone (PCL) and poly (ethylene oxide terephthalate)-poly(butylene terephthalate) (PEOT/PBT) were used as structural biomaterials to form blends with sodium alginate. PCL and PEOT/PBT were selected due to their well-known physico-chemical and mechanical properties as synthetic polymers (Chong et al., 2007; Nandakumar et al., 2010; Gautam et al., 2014), while PEO was selected because it reduces electrical conductivity and surface tension of the alginate solution, leading to fiber formation (Saquing et al., 2013).

The electrospun scaffolds were characterized, considering the fiber morphology, diameter, and inter-fiber pore size. The effect of flow rate, air gap, and scaffold composition were also considered as variables affecting the fiber diameter. Finally, dermal fibroblasts were cultured on both sides of PCL and PCL/Alginate scaffolds to create a dermal substitute.

## Materials and Methods

### Fabrication of ESP Scaffolds

Scaffolds of different compositions were produced using the electrospinning technique. All solutions were prepared and stirred overnight at room temperature before use. In order to produce alginate bare electrospun scaffolds, PEO was used as a sacrificial carrier. Alginate scaffolds were electrospun from a solution containing 1% wt/v alginic acid and 4% wt/v PEO (Sigma-Aldrich, Mw ~8.000.000) in demineralised water. After electrospinning, the alginate scaffolds were crosslinked for 2 h in a 50 mM calcium chloride (CaCl_2_, Merck, Germany) solution, creating stable fibrous gels. The PEO was removed by washing the scaffolds for 24 h in demineralised water.

PCL bare scaffolds were electrospun from 20% wt/v solutions of the polymer (Mv = 42.500, Sigma-Aldrich) in chloroform (CHCl_3_). Similarly, alginate / PCL scaffolds were prepared from solutions containing 20% wt/v PCL and 0.5 or 1% w/v Alginic acid (3.500 cps, Sigma-Aldrich) in chloroform.

PEOT/PBT was obtained from PolyVation (The Netherlands). The copolymer used was 1000PEOT70PBT30, where 1,000 is the molecular weight in g/mol of the starting PEG blocks for the copolymerization, 70 is the weight ratio of PEOT and 30 the weight ratio of PBT domains in the copolymer. PEOT/PBT based scaffolds were electrospun from solutions containing 14% wt/v polymer and 0, 0.5, or 1% wt/v alginic acid in CHCl_3_. The working solution was loaded into a syringe, and the flow rate was controlled using a syringe pump (KDS 100, KD Scientific). A range of flow rates was tested, from 0.05 to 4.5 ml/h, as well as different gaps varying from 15 to 28 cm. A needle with a diameter of 18 gauge was used as a spinneret for all the experiments and, unless indicated, the applied voltage was kept constant at 16 kV. A stainless steel collector, covered by an aluminum foil, was used to collect the spun scaffold. The environmental conditions were kept at 21°C and 35% RH.

### Scaffold Characterization

#### Scanning Electron Microscopy

The morphology of electrospun fibers was observed using a scanning electron microscope (SEM) in secondary electron mode (XL 30 ESEM-FEG, Philips). Prior to imaging, samples were dried on a vacuum oven to remove any remaining solvent. Thereafter, samples were mounted on aluminum stubs and coated with gold using a sputter coater (Cressington SputterCoater 108auto) previous to SEM imaging. Fiber diameters were measured from the SEM photographs by measuring 40 fibers per condition using an image analysis software (Image J, National Institutes of Health, USA). Furthermore, the pore size between adjacent fibers, called inter-fiber pore size, was calculated by measuring 50 different inter-fiber pores.

#### Attenuated Total Reflectance Fourier Transform Infrared Spectroscopy

Attenuated total reflectance Fourier transforms infrared (ATR-FTIR) spectroscopic analysis of electrospun nanofibrous scaffolds was performed to determine the functional groups present in the scaffolds. The nanofibrous mat was peeled off from the aluminum foil after electrospinning, and analysis was performed with a Tensor 27 Bruker, Germany. For each measurement, 32 scans were colected with 4 cm^−1^ resolution.

#### Contact Angle

The water contact angle of the ESP meshes was determined using the sessile drop method. The measurements were performed using a goniometer, Data Physics (model OCA 15 Plus) system, equipped with an electronic syringe, a video camera and SCA 20 software. First, the samples were placed over the plate of the goniometer and centered with the tip of the needle. Then, using the software, a drop of 4 μl of ultra-pure water was released through the needle, over the sample. This procedure was observed by a video camera, allowing the capture of the exact moment when the drop reached the sample surface. This procedure was repeated three times for all the samples in the study with the acquisition of the mean contact angle of the right and left part of the drop.

#### Differential Scanning Calorimetry

Differential scanning calorimetry (DSC) experiments were performed with a nitrogen refrigerated cooling system (TA Instruments) acting at 30 ml/min. Prior to the scans, temperature and energy calibrations were accomplished with an indium standard. Each sample was (a) left at 25°C for 2 min, (b) heated from 25°C to 180°C at 20°C/min, (c) held for 1.0 min at 180.00°C, (d) cooled from 180°C to −50°C at 30°C/min, (e) kept for 2.0 min at −50°C, (f) heated again from −50 to 180°C at 10°C/min, and finally (g) cooled to 25°C at 300°C/min. For the samples of bare alginate, the heating scans were brought to a maximum of 100°C, instead of 180°C, to avoid sample degradation.

### Human Dermal Fibroblast Cell Culture on ESP Scaffolds

#### Culture of HDF

HDFs were purchased from American Type Culture Collection (ATCC, Manassas, VA, USA) and cultured to confluence in DMEM supplemented with 10% heat-inactivated fetal bovine serum (Cambrex), 100 units/L penicillin (Life Technologies), and 10 μg/mL streptomycin (Life Technologies). Culture medium was replaced every 3 days, and the cultures were maintained in a humidified incubator at 37°C with 5% CO_2_. Once the HDFs reached 70% confluence, they were trypsinised and passaged at 1:3 ratios. Fibroblasts at a passage among three–five were used for this experiment. Considering the commercial source of HDFs, ethical approval was not required by the authors.

#### Dual Side Growth on PCL and PCL/Alginate Scaffolds

The electrospun scaffolds chosen for cell culture studies were made from 20% wt/v PCL and 20% wt/v PCL + 1% wt/v alginate. Scaffolds were placed in a cell insert (Figure S1, an area equivalent to a 96-well plate) and sterilized in ethanol for 2 h, rinsed with PBS and soaked in cell culture medium overnight prior to cell seeding. The HDF were seeded on the scaffolds at a cell density of 1 × 10^4^ cells/well and incubated for 7 days. The inserts were then flipped over and incubated for another 7 days with new HDF cells seeded again on the top of the ESP mesh. The culture medium used was changed every 3 days.

#### Cell Behavior on PCL and PCL/Alginate ESP Scaffolds

##### Cell proliferation assay

Cell metabolism was assessed using a Presto blue assay (Biosource, Camarillo, CA, USA) according to the manufacturer's protocol. Briefly, culture medium was replaced with medium containing 10% (v/v) Presto blue solution and the cells were incubated at 37°C for 2 h in the dark. Fluorescence was measured at 590 nm on a Victor3 1,420 Multilabel plate reader. Cell metabolism was analyzed on day 1, 3, 7, 8, 10, 12, and 14 (*n* = 3).

##### Cell viability

Evaluation of cell number was performed after 14 days in culture using the CyQUANT® DNA quantification kit (Invitrogen) according to the manufacturer's protocol. Samples were first digested overnight at 56°C on a 1 mg/mL solution of proteinase K (Sigma-Aldrich) in tris/EDTA buffer (pH 7.5) under vigorous stirring and consequently freeze-thawed three times using liquid nitrogen. For each condition, triplicates of 40 μL of proteinase K digested cell lysate were used for the analysis. Fluorescence was measured at an excitation wavelength of 480 nm and an emission wavelength of 520 nm using a Perkin Elmer LS50B plate reader. To generate a DNA standard curve, serial dilutions of bacteriophage λ DNA (provided with the CyQUANT kit) ranging from 0.0 to 2.0 μg/mL were prepared. A reference standard curve was also created to convert sample fluorescence values into cell numbers. HDF cell suspensions were used with dilutions ranging from 0 to 50,000 cells per sample. For each experiment, a standard calibration curve was generated by plotting measured fluorescence values in these samples vs. cell number, as determined previously from cell suspensions using an hemocytometer.

##### Cell morphology

After culture of HDF on ESP scaffolds cell morphology was evaluated via immunofluorescence. After removal of the medium, the scaffolds were washed with phosphate buffer saline (PBS) and fixed with paraformaldehyde (4% in PBS) for 30 min. Samples were then permeabilised and blocked simultaneously with TBP (0.1% Triton X-100, 1% BSA in PBS) for 1 h at room temperature, and washed with 1% bovine serum albumin (BSA) in PBS for 5 min. Samples were then incubated with fluorescently conjugated primary antibody (1% BSA in PBS), at 4°C overnight (anti-Collagen type I N- terminal, conjugated to ATTO-TEC GmbH 488, Sigma-Aldrich) (Leferink et al., 2018). After incubation, constructs were washed two times with PBST solution (0.1 % Tween-20 in PBS) and once with PBS for 5 min each. Subsequently, Phalloidin Alexa Fluor 594 (Invitrogen) diluted in 1% BSA in PBS (1:40) was added to stain F-actin and incubated for 30 min at RT. Finally, 4′,6′Diamidin-2′-phenylindoldihydrochlorid (DAPI, 100 ng/mL in PBS, Sigma-Aldrich, Munich, Germany) was added and incubated for 5 min, to stain the cell nucleus. After washing three times with PBS, the constructs were removed from the insert and placed in a glass slide. Mowiol 4-88 (Sigma-Aldrich) mounting media was added, and a coverslip was placed on the top of the constructs before letting them dry overnight. Samples were imaged using a Nikon A1 confocal microscope (Nikon Instruments Inc.).

### Statistical Analysis

Results are presented as mean ± SD and compared using either one-way ANOVA (multiple conditions) or Student's *t*-test (two conditions). Statistical significance was set to *p* < 0.05.

## Results

### Scaffold Characterization

**Figure 1:**
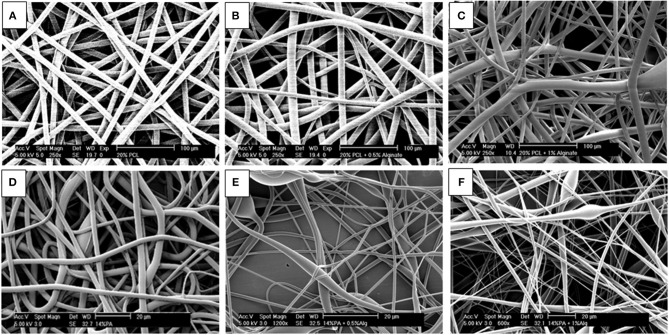
SEM micrographs of electrospun scaffolds made of: **(A)** 20% PCL fibers, **(B)** 20% PCL + 0.5% Alginate fibers, **(C)** 20% PCL + 1% Alginate fibers, **(D)** 14% PEOT/PBT fibers, **(E)** 14% PEOT/PBT + 0.5% Alginate fibers, and **(F)** 14% PEOT/PBT + 1% Alginate fibers. Flow rate: **(A,C,D,E)** 3.5 ml/h; **(B)**1.5 ml/h; **(F)** 2.5 ml/h. Air gap: 25 cm. Scale bar: **(A–C)** 100 μm & **(D–F)** 20 μm.

**Figure 2:**
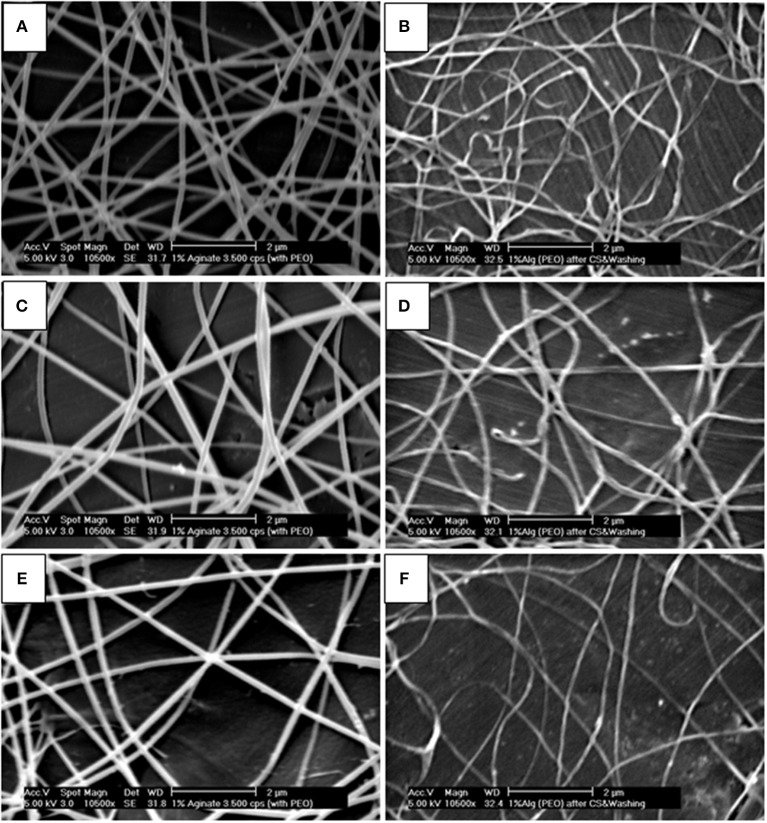
SEM micrographs of 1% Alginate + 4% PEO electrospun scaffolds before and after “cross linking + washing”: **(A)** native scaffold, flow rate of 0.05 ml/h and air gap of 25 cm; **(B)** sample “A” after cross linking and washing; **(C)** native scaffold, flow rate of 0.2 ml/h and air gap of 28.5 cm; **(D)** sample “C” after cross linking and washing; **(E)** native scaffold, flow rate of 0.8 ml/h and air gap of 28.5 cm; **(F)** sample “E” after cross linking and washing. Scale bar: 2 μm.

Figure 1 shows the fiber morphology of the different electrospun scaffolds fabricated in this study. Fibers presented a smooth morphology and varied drastically in size depending on the presence or not of synthetic polymers as carriers of alginate. In order to characterize the alginate electrospun scaffolds in a native form (containing PEO as a carrier) but also after cross-linking and washing, the electrospun samples were imaged two times (Figure 2).

### Effect of Flow Rate and Air Gap on Fiber Diameter of Electrospun Scaffolds

**Figure 3:**
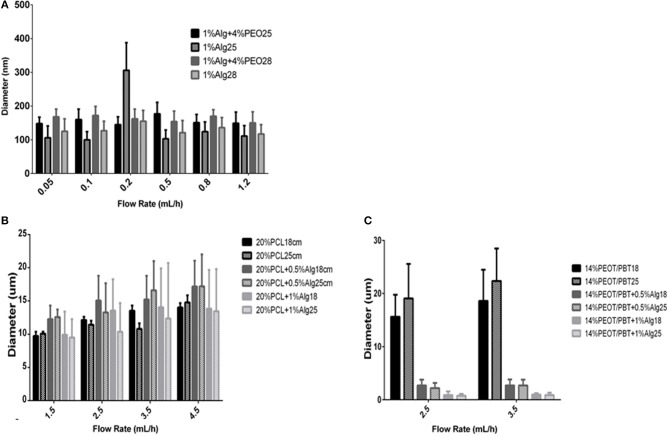
Effect of the flow rate and air gap on fiber diameter of different materials. **(A)** Effect of the flow rate (0.05–1.2 mL/h) and air gap (25 and 28 cm) on fiber diameter of Alginate/PEO scaffolds prepared from 1% Alginate + 4% PEO solutions and the same scaffolds after alginate crosslinking and PEO removal; **(B)** Effect of the flow rate (1.5–4.5 mL/h) and air gap (18 and 25 cm) on fiber diameter of PCL and PCL/Alginate blended electrospun scaffolds (20% PCL, 20% PCL + 0.5% Alginate, 20% PCL + 1% Alginate); **(C)** effect of the flow rate (1.5–4.5 mL/h) and air gap (18 and 25 cm) on fiber diameter of PEOT/PBT and PEOT/PBT/Alginate blended electrospun scaffolds (14% PEOT/PBT, 14% PEOT/PBT + 0.5% Alginate, 14% PEOT/PBT + 1% Alginate).

As shown in Figure 3A, PEO/alginate fiber diameter was characterized after spinning at five different flow rates and at two specific air gaps. Fiber diameter ranged from 145 ± 23 nm to 177 ± 34 nm for an air gap of 25 cm and from 150 ± 33 to 172 ± 27 nm for 28 cm. No significant effect of the flow rate was observed on fiber diameter, while an increase in air gap generally increased the fiber diameter. After alginate cross-linking and washing of PEO, the fiber diameter of the resulting scaffolds decreased (Table S1). Fibers varied from 106 ± 35 to 306 ± 82 nm. No specific trend in fiber diameter variations could be detected with varying flow rate or air gap in the range here considered.

Alginate was crosslinked using CaCl_2_ solution. Some examples of the morphology and dimensions of the fibers, for different electrospun scaffolds before and after crosslinking and washing are shown in Figure 2. Comparing the results of the diameter of the fibers before and after washing, it is possible to conclude that the alginate electrospun fibers in native form were higher likely due to the presence of PEO, with an exception for the result corresponding to 0.2 ml/h and 25 cm.

Upon electrospinning of 20% PCL solutions, the fiber diameter increased from 9.72 ± 0.64 to 14.02 ± 0.66 μm by increasing the flow rate from 1.5 to 4.5 ml/h. An increase in the air gap from 15 to 25 cm generally resulted in no variations or a slight decrease in fiber diameter (Figure 3B).

With the addition of alginate to the 20% PCL solution, the fiber diameter increased when the flow rate was increased for both 18 and 25 cm air gaps. Increasing the alginate content in the solution, the fiber dimensions of the fabricated scaffolds decreased, and scaffolds were comprised of a more heterogeneous population of fibers. No significant variations were observed when changing the air gap. Fiber diameter varied from 12.26 ± 2.04 to 17.2 ± 4.8 μm when 0.5% alginate was added, and from 9.5 ± 2.75 to 13.83 ± 5.83 μm when 1% alginate was included in the PCL solution (Table S3).

PEOT/PBT electrospun scaffolds presented a fiber diameter that varied from 15.62 ± 4.14 to 18.61 ± 5.86 μm, for a working distance of 18 cm, and from 19.06 ± 6.51 to 22.34 ± 6.13 μm, for 25 cm, with increasing flow rate and air gap (Figure 3C). Interestingly, when PEOT/PBT was combined with a 0.5% solution of alginate, the fiber diameter was drastically reduced. A further increase in alginate content to 1% resulted in a further decrease of fiber dimensions from 2.67 ± 1.10 to 0.876 ± 0.69 μm (Table S3).

### Effect of Flow Rate and Air Gap on Scaffold Inter-Fiber Pore Size

**Figure 4:**
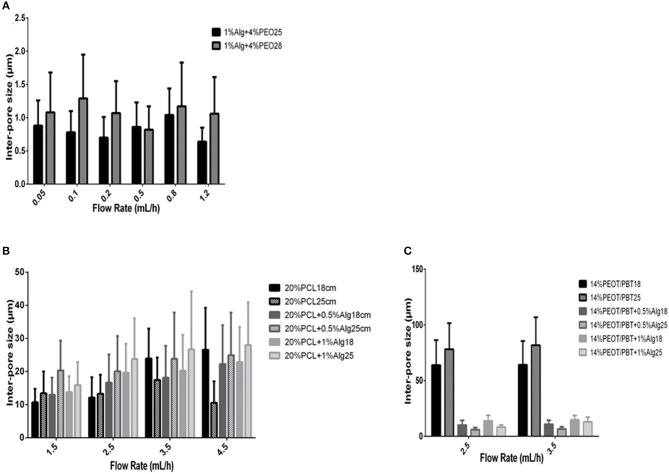
Effect of the flow rate and air gap on inter-pore size of different materials. **(A)** Effect of the flow rate (0.05–1.2 mL/h) and air gap (25 and 28 cm) on inter-pore size of Alginate/PEO blended electrospun scaffolds after crosslinking and washing with calcium chloride (1% Alginate + 4% PEO); **(B)** effect of the flow rate (1.5–4.5 mL/h) and air gap (18 and 25 cm) on inter-pore size of PCL and PCL/Alginate blended electrospun scaffolds (20% PCL, 20% PCL + 0.5% Alginate, 20% PCL + 1% Alginate); **(C)** effect of the flow rate (1.5–4.5 mL/h) and air gap (18 and 25 cm) on inter-pore size of PEOT/PBT and PEOT/PBT /Alginate blended electrospun scaffolds (14% PEOT/PBT, 14% PEOT/PBT + 0.5% Alginate, 14% PEOT/PBT + 1% Alginate).

In case of PEO/Alginate scaffolds, the inter-pore size generally increased when the air gap increased and ranged from 0.70 ± 0.31 to 1.29 ± 0.66 μm (Table S2). No specific influence of flow rate variations was observed on inter-fiber pore size (Figure 4A).

Generally, for the 20% PCL solution, the inter-fiber pore size increased with the flow rate, as well as with a larger air gap. Pore size varied from a minimum of 10.68 ± 4.11 μm to a maximum of 26.57 ± 12.69 μm for the flow rates and air gaps analyzed (Figure 4B). The same trend was observed when adding alginate to the PCL solution. Furthermore, the addition of alginate to the solution usually resulted in an increase of the inter-fiber pore size. An increase in alginate content also contributed to increasing the inter-fiber pore size. Pore size varied from 12.96 ± 5.19 to 24.92 ± 12.90 μm with the addition of 0.5% alginate, and from 13.79 ± 4.79 to 26.7 ± 17.52 μm when alginate content was increased to 1% (Table S3).

Electrospining of 14% PEOT/PBT solutions resulted on an increased inter-pore size when the air gap increased, while no consistent influence of the flow rate could be observed. When alginate was added, the inter-fiber pore size drastically decreased (Figure 4C). For example, the pore size changed from 63.90 ± 22.45 μm for 14% PEOT/PBT to 10.16 ± 4.10 μm for 14% PEOT/PBT with 0.5% alginate at a flow rate of 2.5 ml/h and an air gap of 18 cm. With increasing alginate content, the pore size appeared to increase (Table S3).

### Physico-Chemical Characterization of Electrospun Meshes

**Figure 5:**
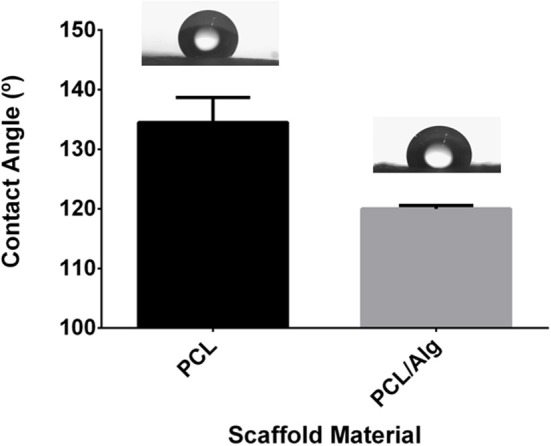
Contact angles of water on PCL and PCL/Alg ESP scaffolds (*T*-test, *P* = 0.0251, there is a significant difference) Photograph of the drop in the exact moment that it reached the sample surface **(left)** PCL **(right)** PCL/Alg ESP mesh.

Substratum wettability is often evaluated by measuring the angle of contact between a liquid of known surface tension and a solid surface (Wiencek and Fletcher, 1997). The water contact angles measured for 20% wt/v PCL and 20% wt/v PCL/1% wt/v alginate are given in Figure 5. This dissimilarity between the samples was noticeable also visually, as the drop of water was more disperse over the PCL/Alg surfaces (Figure 5). Both materials had contact angles higher than 90°, resulting in hydrophobic surfaces. However, the presence of alginate in the scaffolds decreased the contact angle compared to the bare PCL.

Differential Scanning Calorimetry (DSC) was used to analyze the thermal behavior and associated transitions of the polymers and blends thereof. Figure S2 shows DSC traces for alginate alone (Figure S2A) or when blended with PEOT/PBT and PCL. DSC traces of alginate shows a characteristic broad endothermic peak with onset at around 60°C that is generally ascribed to loosely bound water molecules with no further transitions observed during cooling or second heating cycles. Thermal transitions of both PCL and PEOT/PBT were unaltered upon blending with alginate. Blends of alginate with PCL showed the characteristic melting peak, at ~55°C, of PCL that was also observed in neat PCL controls (Figure S2B). A consecutive cooling cycle on both neat and blended PCL, resulted on the crystallization of the polymer and the associated exothermic peak at around 45°C. Similarly, DSC traces of alginate PEOT/PBT blends show the characteristic endothermic peak of alginate at 100°C (onset at 60°C). During the first heating scan a small endothermic transition was detected at a temperature of 160°C, probably corresponding to the melting peak of PEOT/PBT. Upon cooling, both neat PEOT/PBT and its alginate blend, showed an exothermic transition characteristic of a crystallization and a glass transition temperature on the second heating cycle, corresponding to the PEOT/PBT polymer (Figure S2C).

### Cell Behavior on PCL and PCL/Alginate ESP Scaffolds

**Figure 6:**
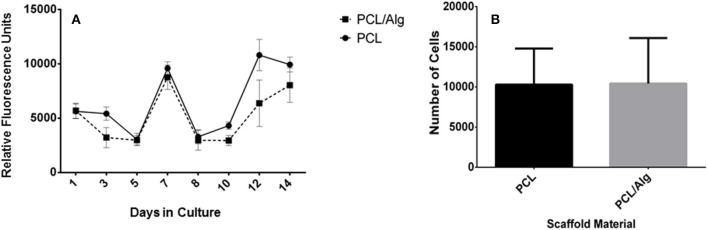
Cell proliferation on PCL and PCL/Alginate ESP Scaffolds. **(A)** Metabolic activity of the cells on PCL and PCL/alginate meshes over 14 days of culture measured by presto blue assay. **(B)** Cell number after 14 days of culture. Data are represented as mean ± standard deviation (*N* = 5; There is no significant difference).

Initial cell seeding on ESP scaffolds resuted on a decrease in metabolic activity of the cells after the first and the second cell seeding (Figure 6A), while in both cases the metabolic activity started to recover after day 5 of culture. At day 7, when the second layer of cells was seeded, the metabolic activity rose and followed again the same patter, with 5 days of reduced activity followed by a later increase. Comparing the PCL and PCL/Alg scaffolds in days 1 and 8, 1 day after each seeding step, it was not possible to note a difference in cell activity. However, as the culture days proceed, PCL had higher values that might be explained by either an increment in metabolic activity or a higher proliferation rate, traduced in a higher cell number.

CyQUANT® DNA assay was used to measure DNA content and cell number on the electrospun scaffold after 14 days in culture (Figure 6B). DNA quantification showed that there is no significant difference between the cell number on PCL and PCL/Alginate scaffolds, implying that the high intensity values measured were a result of a higher metabolic activity.

**Figure 7:**
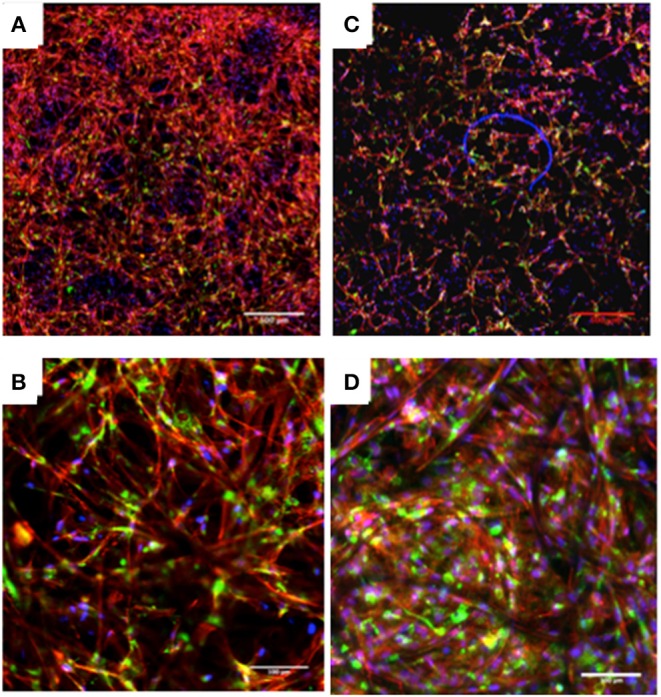
Confocal images of hDF cultured in PCL **(A,B)** and PCL/Alginate **(C,D)** electrospun scaffolds after 14 days in culture showing collagen type I staining (Blue—DAPI, Red—Phalloidin, Green—Collagen I). Collagen type I is found in both conditions. Scale bar: **(A,C)** 500 μm; **(B,D)** 100 μm.

Collagen type I probes are usually applied on culture systems to monitor and characterize tissue formation in 3D scaffolds. Cell morphology and Collagen I production was assessed by confocal microscopy as shown in Figure 7. hDFs cultured on ESP meshes presented an even distribution. Collagen I production was observed as fibrillary structures on both PCL and PCL/Alginate electrospun scaffold after 14 days in culture. To conclude, there was not a distinct difference in collagen production between PCL electrospun fibers (Figures 7A,B) and PCL/Alginate meshes (Figures 7C,D).

## Discussion

Electrospun scaffolds were produced using different flow rates and air gaps in order to investigate the effect of these parameters on fiber diameter and scaffold inter-fiber pore size. Generally, it is known that with increasing flow rate, there is an increase of fiber diameter; with an increasing air gap, there is a reduction in fiber diameter (Geng et al., 2005; Ki et al., 2005; Zhao et al., 2005). However, this effect is not evident in all cases as some experiments showed that the fiber diameter does not change significantly when the air gap changes once the solvent has time to evaporate before reaching the target.

The addition of PEO was critical to obtain alginate electrospun scaffolds, as alginic acid solutions only resulted in no deposition on the collector, likely due to the anionic nature of the polymer. Addition of PEO further increased the viscosity of the solution and affected the surface tension leading to the formation of fibers, as proposed by Doshi and Reneker (1995). Adding a cross-linking solution to the alginate scaffolds promotes the creation of inter-chain bridges that causes gelling of alginate. Synthetic polymers such as PCL and PEOT/PBT were also spun alone and blended with alginate.

Figure 1, shows the formation of fibers with heterogeneous diameters due to the presence of two fiber populations. With the addition of 1% alginate (Figures 1C,F) to the polymer solution, there was an increase in the thinner fiber population compared to the 0.5% blends (Figures 1B,E). Thinner fibers are rare on non-blended polymers (Figures 1A,C). Our results also show a decrease in PEO/Alginate fiber diameter after cross-linking and washing steps (Figure 2).

In general, the fiber diameter increased when the distance between the spinneret and the collector increased. However, a relation between the fiber diameter and the flow rate is not well-known since it can increase or decrease with increasing the flow rate. Comparing the fiber diameter of alginate scaffolds before and after cross-linking and washing off the PEO (Figures 2, 3A) showed a reduction on the fiber diameter. Moreover, the fiber diameters measured for the uncross-linked fibers were higher, in contrast to those ones reported by Shields et al. (2004) who studied cross-linked and non-cross-linked collagen electrospun scaffolds using glutaraldehyde and found that cross-linking increases the fiber diameter as well as the thickness of the scaffolds. It is also important to refer that, in this case, alginate solutions contained PEO as a carrier system for electrospinning. When the fabricated scaffolds were placed in an alginate aqueous cross-linking solution the alginate is expected to shrink as a result of the tighter polymer network formed and PEO dissolved and leached out. The combination of these phenomena might explain why the fibers have a reduced diameter after this process.

Electrospun scaffolds had similar fiber dimensions for all the different air gaps considered. According to Geng et al. (2005) and some other authors (Ki et al., 2005; Zhao et al., 2005), the distance plays an important role in order to determine fiber and beads generation. As shown in Figure 3, it was challenging to set a relation between fiber diameter and flow rate, as well as with the air gap. These results add on Fong et al. (1999), Ki et al. (2005) and Zhao et al. (2005) which concluded that the effect of the air gap on fiber size is not as significant as other parameters as soon as the solvent has time to evaporate. As for the PEO fibers, for the PCL blends (Figures 3A,B) was hard to find a tendency in the effect of the air gap in the fiber diameter. However, regarding the effect of the flow rate, there is a general increase in fiber diameter when there is more polymer extruded through the needle as expected. Curiously, for the hybrids 20%PCL + 0,5% alginate and 20%PCL + 1% alginate, the fiber diameter was reduced with the increasing of the air gap. It was also observed that 20%PCL and 20%PCL + 1%Alginate had similar values of fiber diameter, while 20%PCL + 0,5%Alginate presented the highest dimensions. The PEOT/PBT scaffolds had smaller diameters in the presence of alginate (Figure 3C).

The inter-fiber pore size was measured for the different scaffolds (Figure 4). In general, the inter-fiber pore size increased when the air gap increased as PEO/Alginate fibers revealed (Figure 4A). However, it was difficult to set a relation between inter-fiber pore size and the flow rate, since it increased or decreased by increasing the flow rate for both air gaps. On the other hand, for 20% PCL scaffold and 20% PCL blended with different percentage of alginate scaffolds (Figure 4B), the inter-fiber pore size generally increased when the flow rate and air gap increased. Concerning 14% PEOT/PBT scaffold, there is a significant decrease in inter-pore size when in the presence of alginate, as expected after the analysis of the fiber diameter (Figure 4C).

Differential scanning calorimetry monitors heat effects associated with phase transitions as a function of temperature. PCL has a melting point of around 55–70°C (depending on the molecular weight) and a glass transition temperature of −70°C approximately (Deschamps et al., 2002). For 20% PCL and 20% PCL + 1% alginate endothermal transitions were observed for both heating scans, as expected for the melting point of this synthetic polymer. Correspondingly, two exothermic transitions were also observed for the bare PCL and its alginate blend during the cooling cycle, corresponding to the crystallization of the polymer (Figure S2).

The melting point of PEOT/PBT is around 150–160°C depending on the molecular weight of the hard segments. DSC traces of both, PEOT/PBT and its alginate blend showed a small melting transition at around 160°C that was only visible during the first heating cycle, probably due to the fast cooling cycle applied that hindered the crystallization of the polymer. The presence of an endothermal transition was also observed at temperatures of around 17°C for PEOT/PBT and its blends, which can be ascribed to a glass transition. In both blends, PCL/alginate and PEOT/PBT/alginate, the presence of the alginate was corroborated as an endothermic transition on the first heating cycle at 100°C that is generally ascribed to loosely bound water molecules to the alginate chains.

Porosity is seen as an important parameter for TE scaffolds. The porous nature of the nanofibrous structures is beneficial for cell infiltration and proliferation. For skin reconstitution, gas and nutrient exchange is of extreme importance for wound healing. To establish a 3D scaffold for wound healing purposes populated by fibroblasts, pores have to be big enough for the cells to migrate into the structure.

Looking at the inter-pore size graphs (Figure 4), it is possible to note that only PCL and PCL blended with Alginate scaffolds had pores within the range of fibroblast cells, which are of sizes 10–100 μm. This was one of the reasons why these scaffolds were selected for cell work (Chong et al., 2007). In our study, cells were cultured on both sides (Figure S1) to increase the cell loading capacity and allow them to proliferate from both ends. Other authors have suggested the use of PCL modified scaffolds as promising candidates for skin tissue engineering. PCL/Gelatin with or without Collagen I are a good example (Gautam et al., 2014).

Further scaffold characterization tests were performed on the selected structures before cell culture. Wettability is a critical factor for cell adhesion, as well as for protein adsorption (Wiencek and Fletcher, 1995). The smaller the contact angle, the higher the interaction of the liquid with functional groups on the substratum surface. Both the type and molecular topography (configuration) of functional groups on the substratum surface affect wettability. In some cases, however, differences in the composition of the substratum surface are not necessarily reflected in the substratum wettability (Wiencek and Fletcher, 1997). Both materials had contact angles resulted in hydrophobic surfaces (Figure 5). However, the presence of alginate in the scaffolds decreased the contact angle compared to PCL alone. Nevertheless, the scaffolds were still hydrophobic, so suitable for serum protein adhesion and subsequent cell culture (Graf and Kappl, 2006).

ATR-FTIR was also performed in order to analyze the composition of the fibers (data not shown). However, it was not possible to detect differences between the PCL and PCL/Alg spectra, probably due to the small amount of alginate introduced to the system.

Metabolic activity decreased after both cell seedings. This might be explained by the fact that, after cell seeding, the confluency of cells over the scaffold produces space stress on the cells and depletion in nutrients supply as previously observed by Gautam et al. (2014). The faster increase of the metabolic activity after the second seeding can be attributed to the support of the cells already adhered to the scaffold from the first seeding.

Although alginate is a polysaccharide to which cells are unable to adhere since it is extremely hydrophilic and does not absorb serum proteins, the fact that it was not observed in the surface of the scaffold might explain the reason why there was no significant difference between the PCL and PCL blended with alginate scaffold (Jeong et al., 2010).

Collagen is the most abundant protein in mammals and of uttermost importance to understand tissue morphogenesis in scaffolds. Collagen fibers provide the tensile strength that allows organs such as the skin to form organized structures. Collagen type I is the most common and forms the bulk of skin collagen, accounting for over 80% of the dermis (Park et al., 2009).

To further examine the morphologies of the cells on the nanofibers, after 14 days in culture, the cells were stained with phalloidin to observe F-actin organization, Collagen type-I to monitor tissue formation, and counterstained with DAPI to visualize the nuclei. Collagen type-I was found in both samples, as seen in Figure 7. In both structures, collagen type-I was distributed over the areas where cells were located, and no notorious difference was found between the PCL and PCL blended with Alginate scaffolds.

## Conclusions

In this study, synthetic, natural and hybrid fibrous scaffolds were successfully produced via electrospinning and its morphology, physicochemical properties as well as the cell response to these materials was characterized. It was investigated whether it is possible to use alginate to produce electrospun fibers, either with a carrier like PEO or blended with different polymers as PCL and PEOT/PBT.

Based on the evidence currently available, it is possible to conclude that hybrid electrospun fibers were successfully produced in the presence of alginate, and that was possible to tune their properties by varying the different parameters. However, further cell work will be needed in order to prove that the presence of alginate improves the biological response of hDF in PCL/Alginate fibers when compared with non-blended PCL fibers. Nonetheless, this study demonstrates that alginate incorporation in ESP fibers is viable and useful for future works.

## Data Availability Statement

All datasets generated for this study are included in the manuscript/Supplementary Files.

## Author Contributions

AG, IO, AL, and LM designed the study and the experiments. AG and IO performed the experiments. SC-E contributed to the data analysis. All authors wrote the manuscript and critically analyzed and revised the data.

### Conflict of Interest

The authors declare that the research was conducted in the absence of any commercial or financial relationships that could be construed as a potential conflict of interest.
