# Supplementary material for: Hybrid Polyester-Hydrogel Electrospun Scaffolds for Tissue Engineering Applications
Source: Front Bioeng Biotechnol. 2019 Sep 25;7:231. doi: 10.3389/fbioe.2019.00231 (PMC6798037; doi:10.3389/fbioe.2019.00231)
Supplement: Supplementary file 1 [file Data_Sheet_1.PDF]

Supporting Information

Hybrid Polyester-Hydrogel electrospun scaffolds for tissue engineering applications

Ana Rita Goncalves de Pinho[1], Ines Odila[1], Anne Leferink[1], Clemens van Blitterswijk[1,2], Sandra Camarero-Espinosa[2] and Lorenzo Moroni[1,2]

[1]Institute for BioMedical Technology and Technical Medicine (MIRA), Tissue Regeneration Department, University of Twente, P.O. Box 217, 7500 AE Enschede, The Netherlands.

[2]MERLN Institute for Technology-inspired Regenerative Medicine, Complex Tissue Regeneration Department, Maastricht University, P.O. Box 616, 6200MD Maastricht, The Netherlands

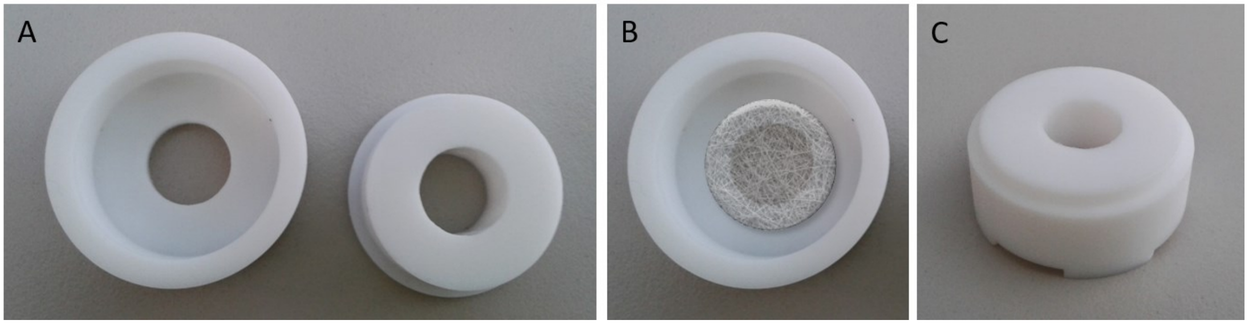

Figure S1. Cell culture inserts – sample insertion procedure: (A) open, (B) insert electrospun mesh, (C) close insert.

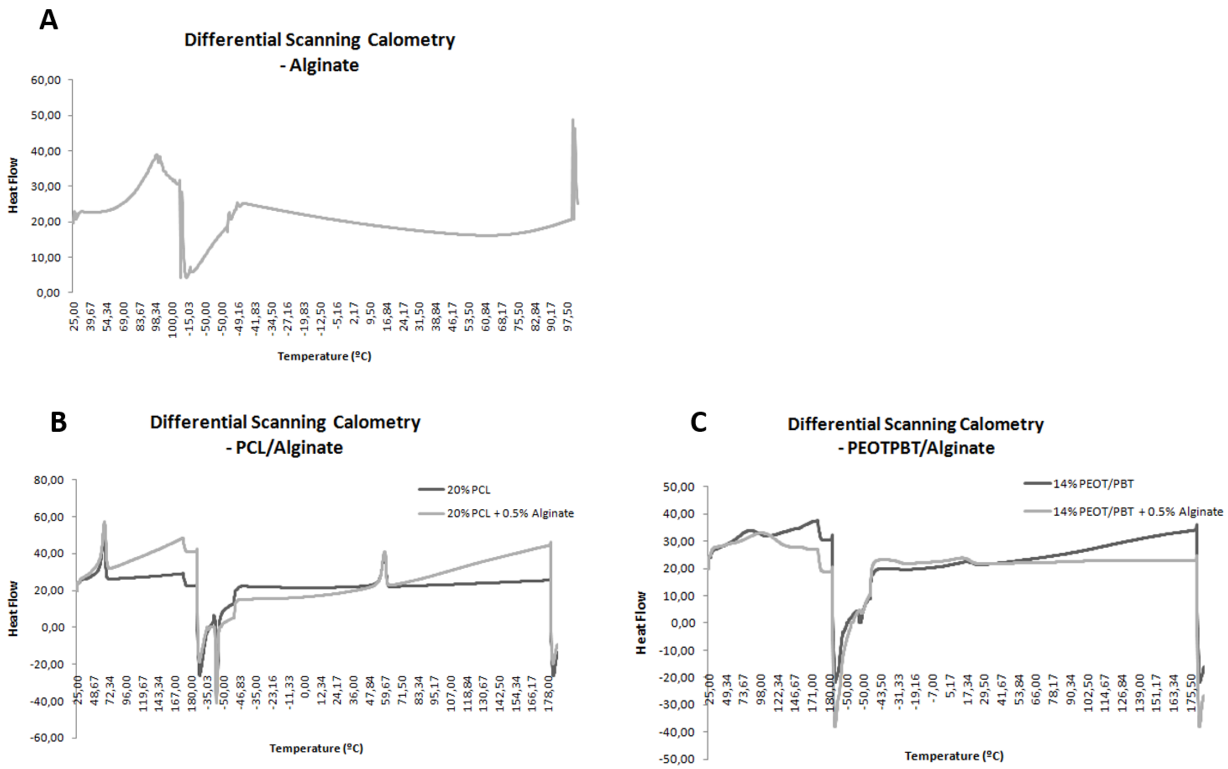

Figure S2. Differential scanning calorimetry traces of (A) 1% Alginate + 4% PEO (B) 20% PCL and 20% PCL + 0.5%, (C) 4% PEOT/PBT and 14% PEOT/PBT + 0.5%.

Polymer Composition	Flow Rate (ml/h)	Fiber Diameter (nm)		After cross linking and washing	Fiber Diameter (nm)	
		Air gap - 25 cm	Air gap - 28 cm		Air gap - 25 cm	Air gap - 28 cm
1% Alginate with 4% PEO	0.05	148 ± 19	168 ± 23		106 ±35	125 ± 37
	0.1	160 ± 31	172 ± 27		100 ± 24	127 ± 28
	0.2	145 ± 23	162 ± 29		306 ± 82	155 ± 32
	0.5	177 ± 34	154 ± 31		103 ± 26	121 ± 36
	0.8	151 ± 24	170 ± 19		124 ± 29	136 ± 30
	1.2	149 ± 33	150 ± 33		111 ± 31	117 ± 28

Table S1 – Alginate/PEO blended electrospun scaffold characterization at different flow rates and air gaps: fiber diameter before and after “cross linking and washing”.

Polymer Composition	Flow Rate (ml/h)	Pore size (µm)	
		Air gap - 25 cm	Air gap - 28 cm
1% Alginate with 4% PEO	0.05	0.88 ± 0.38	1.08 ± 0.60
	0.1	0.78 ± 0.32	1.29 ± 0.66
	0.2	0.70 ± 0.31	1.07 ± 0.48
	0.5	0.86 ± 0.37	0.82 ± 0.35
	0.8	1.04 ± 0.40	1.17 ± 0.66
	1.2	0.64 ± 0.21	1.06 ± 0.55

Table S2 – Inter-pore size of Alginate/PEO blended electrospun scaffolds at different flow rates and air gaps.

Polymer Composition	Air Gap (cm)	Fiber Diameter (µm)				Inter-pore size (µm)			
		Flow rate - 1.5 ml/h	Flow rate - 2.5 ml/h	Flow rate - 3.5 ml/h	Flow rate - 4.5 ml/h	Flow rate - 1.5 ml/h	Flow rate - 2.5 ml/h	Flow rate - 3.5 ml/h	Flow rate - 4.5 ml/h
**20% PCL**	15	-	12.18 ± 1.27	-	-	-	24.69 ± 11.87	-	-
	18	9.72 ± 0.64	12.13 ± 0.5	13.52 ± 0.80	14.02 ± 0.66	10.68 ± 4.11	12.13 ± 6.14	23.91 ± 9.02	26.57 ± 12.69
	20	-	12.45 ± 0.43	-	-	-	11.86 ± 4.84	-	-
	25	10.07 ± 0.31	11.43 ± 0.59	10.79 ± 0.85	14.77 ± 1.06	13.42 ± 6.62	13.26 ± 5.79	17.44 ± 6.77	10.55 ± 6.48
**20% PCL + 0.5% Alginate**	18	12.26 ± 2.04	15.07 ± 3.72	15.21 ± 3.57	17.18 ± 3.86	12.96 ± 5.19	16.65 ± 8.48	18.13 ± 9.60	24.92 ± 12.90
	25	12.57 ± 1.14	13.27 ± 4.39	16.60 ± 4.39	17.2 ± 4.8	20.34 ± 9.02	20.05 ± 10.69	23.86 ± 13.94	22.84 ± 10.63
**20% PCL + 1% Alginate**	18	9.90 ± 3.51	13.55 ± 4.70	14.06 ± 5.85	13.83 ± 5.83	13.79 ± 4.79	19.64 ± 8.76	20.25 ± 10.83	27.99 ± 12.96
	25	9.50 ± 2.75	10.36 ± 4.31	12.35 ± 8.37	13.46 ± 6.35	15.93 ± 6.92	23.78 ± 12.31	26.70 ± 17.52	
**14% PEOT/PBT**	18	-	15.62 ± 4.14	18.61 ± 5.86	-	-	63.90 ± 22.45	64.17 ± 21.29	-
	25	-	19.06 ± 6.5	22.34 ± 6.13	-	-	78.08 ± 23.43	81.73 ± 25.14	-
**14% PEOT/PBT + 0.5% Alginate**	18	-	2.67 ± 1.10	2.69 ± 1.13	-	-	10.16 ± 4.10	10.82 ± 3.67	-
	25	-	2.18 ± 0.97	2.67 ± 1.10	-	-	5.93 ± 1.97	6.60 ± 1.92	-
**14% PEOT/PBT + 1% Alginate**	18	-	0.876 ± 0.690	0.972 ± 0.259	-	-	13.93 ± 4.89	14.79 ± 3.99	-
	25	-	0.742 ± 0.343	0.880 ± 0.440	-	-	8.32 ± 1.90	13.01 ± 4.27	-

Table S3 – Effect of flow rate and air gap on fiber diameter and inter-fiber pore size, considering different compositions of electrospun scaffolds (20% PCL, 20% PCL + 0,5% Alginate, 20% PCL + 1% Alginate & 14% PEOT/PBT, 14% PEOT/PBT + 0,5% Alginate, 14% PEOT/PBT + 1% Alginate). * In this case, just 20-30 inter-pore size was measured due the absence of more data. ** Absence of data, due to the lack of information.
